# Iron Bioavailability Studies of the First Generation of Iron-Biofortified Beans Released in Rwanda

**DOI:** 10.3390/nu9070787

**Published:** 2017-07-21

**Authors:** Raymond Glahn, Elad Tako, Jonathan Hart, Jere Haas, Mercy Lung’aho, Steve Beebe

**Affiliations:** 1 USDA-ARS Robert Holley Center for Agriculture and Health, Ithaca, NY 14853, USA; elad.tako@ars.usda.gov (E.T.); jon.hart@ars.usda.gov (J.H.); 2 Division of Nutritional Sciences, 220 Savage Hall, Cornell University, Ithaca, NY 14853, USA; jdh12@cornell.edu; 3 International Center for Tropical Agriculture (CIAT), Regional Office for Africa, P.O. Box 823-00621, Nairobi 00100, Kenya; m.lungaho@cgiar.org; 4 International Center for Tropical Agriculture (CIAT), Km 17, Recta Cali–Palmira CP 763537, Apartado Aéreo 6713, Cali, Colombia; s.beebe@cgiar.org

**Keywords:** beans, *Phaseolus vulgaris*, iron, bioavailability, biofortification

## Abstract

This paper represents a series of in vitro iron (Fe) bioavailability experiments, Fe content analysis and polyphenolic profile of the first generation of Fe biofortified beans (*Phaseolus vulgaris*) selected for human trials in Rwanda and released to farmers of that region. The objective of the present study was to demonstrate how the Caco-2 cell bioassay for Fe bioavailability can be utilized to assess the nutritional quality of Fe in such varieties and how they may interact with diets and meal plans of experimental studies. Furthermore, experiments were also conducted to directly compare this in vitro approach with specific human absorption studies of these Fe biofortified beans. The results show that other foods consumed with beans, such as rice, can negatively affect Fe bioavailability whereas potato may enhance the Fe absorption when consumed with beans. The results also suggest that the extrinsic labelling approach to measuring human Fe absorption can be flawed and thus provide misleading information. Overall, the results provide evidence that the Caco-2 cell bioassay represents an effective approach to evaluate the nutritional quality of Fe-biofortified beans, both separate from and within a targeted diet or meal plan.

## 1. Introduction

Studies on iron (Fe) biofortification of the common bean were published as early as 2000, approximately a year or two before the term “biofortification” was coined [1]. Prior to 2000, experiments were primarily conducted in rodent models using intrinsically labelled crops, or in humans with extrinsically labelled foods and meals [2]. The cost and limitations of such in vivo studies often prevented the experimental approach from addressing important aspects of bean Fe bioavailability, such as the effects of polyphenols, phytate, and the influence of other foods consumed with beans. Since then, advances have been achieved that allow for more extensive screening of foods and development of staple food crops and food products for improved nutritional quality of Fe. The coupling of the in vitro digestion techniques with Caco-2 cell monolayers was a significant advance that enabled direct in vitro examination of factors that influence Fe bioavailability [3]. This approach utilizes Caco-2 cell ferritin formation as a marker of Fe uptake, which enables scientists to avoid the cost and methodological issues associated with isotopic labelling. Moreover, the ferritin formation approach is a sensitive, cost-effective marker of Fe uptake that dramatically increased the throughput of the system. As a result, this in vitro model has demonstrated the potential to be a tool that can be effectively coupled with modern plant breeding approaches to identify regions of the genome that can influence Fe bioavailability and identify varieties and processing effects that warrant further pursuit to increase the nutritional quality of Fe [4,5,6]. When this in vitro approach is further coupled with an established animal model of Fe bioavailability it represents an effective approach to refine the experimental approach for human studies [7]. This combination of in vitro and in vivo animal studies can also be used to address issues not feasible to conduct in human trials.

The present study is an excellent example of how the established Caco-2 cell bioassay can be applied to evaluate key nutritional factors that could influence the effectiveness of Fe-biofortified beans. Studies were designed to reflect bean consumption in the context of typical meals of Rwanda, and more specifically to compare meals used in human absorption trials and efficacy studies which used the same exact varieties and harvests of beans [8,9]. The objective of this work was to demonstrate how the Caco-2 cell bioassay for Fe bioavailability can be applied to thoroughly and cost-effectively evaluate Fe-biofortified crops prior to a human study or release in the food system. Where appropriate, direct comparisons of the in vitro results to parallel or similar human studies are presented and critically evaluated.

## 2. Materials and Methods

### 2.1. Chemicals, Enzymes, and Hormones

All chemicals, enzymes, and hormones were purchased from Sigma Chemical Co. (St. Louis, MO, USA) unless stated otherwise.

### 2.2. Food Samples and Preparation

The normal and high Fe beans are the same varieties, and the same harvests were used for an animal feeding trial and from a parallel a human efficacy trial in Rwanda [8,10]. These bean lines are best described as “cream seeded carioca” varieties. Approximately 15 kg of the normal and high Fe varieties were available, and subsamples were taken from this stock for the various studies presented herein. For all studies, the bean samples were not pre-soaked, cooked by autoclave for 15 min in a 3:1 volume of water:bean, then freeze-dried and ground into powder with a common coffee grinder. 

The menu items from the human efficacy study, reported in Experiment 1, were samples taken directly from the serving line used in the human study in Rwanda [8]. The samples were freeze dried for shipment to the lab of the first author for assay. For the in vitro Fe bioavailability assay, 0.5 g of the lyophilized sample were used for each replicate of the assay, as per the in vitro digestion procedure described below.

The potato sample used in the results presented in Experiment 2 was an organically grown Idaho white potato purchased at a local supermarket that was peeled and cooked by autoclave, then ground to a powder. It was deemed to be nutritionally similar to the white potato served in the parallel human trials. The rice sample used in this figure was polished, and of the Nishiki variety, and purchased at a local supermarket. These test meals were designed to match the relative combinations of beans and rice or beans and potato used in a similar human study. However, it is important to note that in the results presented in Experiment 2, the additional amount of Fe added as a stable isotope in the human study was not included in this in vitro study. This fact is important, as studies have shown, that the primary assumption of extrinsic labelling studies (i.e., complete isotopic exchange and equilibration of the extrinsic isotope) may not occur [11]. This point will be addressed in the discussion. 

In the results presented in Experiments 3 and 4, extrinsic Fe the form of ^58^Fe was added to the bean sample in the exact same relative ratio as done in similar human studies [9]. However, unlike the human study, potato or rice were not included. The ratio was 0.4 mg ^58^Fe per 55 g of beans (dry weight). At the in vitro level, these amounts correspond to 0.5 g of dry ground bean sample plus 4 µg ^58^Fe.

### 2.3. Iron Content of Food Samples, and Caco-2 Cells

Dried, ground food samples (0.5 g) were treated with 3.0 mL of 60:40 HNO_3_ and HClO_4_ mixture into a Pyrex glass tube and left for overnight to destroy organic matter. The mixture was then heated to 120 °C for two hours and 0.25 mL of 40 µg/g Yttrium added as an internal standard to compensate for any drift during the subsequent inductively coupled plasma atomic emission spectrometer (ICP-AES) analysis. The temperature of the heating block was then raised to 145 °C for 2 h. If necessary, more nitric acid (1–2 mL) was added to destroy the brownish color of the organic matter. Then, the temperature of the heating block raised to 190 °C for ten minutes and turned off. The cooled samples in the tubes were then diluted to 20 mL, vortexed and transferred onto auto sampler tubes to analyze via ICP-AES. The model of the ICP used was a Thermo iCAP 6500 series (Thermo Jarrell Ash Corp., Franklin, MA, USA). For the measurement of ^58^Fe isotopes in Caco-2 cells, cell isolates were treated as described above, and ^58^Fe was quantified using inductively coupled plasma mass spectrometry (Agilent Model 7500CS, Agilent Technologies, 5301 Stevens Creek Blvd, Santa Clara, CA 95051, USA).

### 2.4. Phytic acid Content of Food Samples

Phytic acid content was measured as phosphorous released by phytase and alkaline phosphatase via a colorimetric assay kit (K-PHYT 12/12, Megazyme International, Wicklow, Ireland). 

Phytate degradation was conducted via the methods of Petry et al. [9]. The maximal effect achievable was a 66% decrease in total phytate phosphorous. To make the “68%” phytic acid samples, a non-treated sample was mixed 50:50 with sample treated for maximal phytate degradation (i.e., 34% phytate).

### 2.5. Polyphenol Analysis of Bean Samples

Seed coat extracts and polyphenol standards were analyzed with an Agilent 1220 Infinity UPLC coupled to an Advion Expression L compact mass spectrometer (CMS). Two (2) μL samples were injected and passed through an Acquity UPLC BEH Shield RP18 1.7 μm 2.1 × 100 mm column (Waters) at 0.35 mL/min. The column was temperature-controlled at 45 °C. The mobile phase consisted of water with 0.1% formic acid (solvent A) and acetonitrile with 0.1% formic acid (solvent B). Polyphenols were eluted using linear gradients of 86.7% to 77.0% A in 0.5 min, 77.0% to 46.0% A in 5.5 min, 46.0% to 0% A in 0.5 min, hold at 0% A for 3.5 min, 0% to 86.7% A in 0.5 min, and hold at 86.7% A for 3.5 min for a total 14 min run time. From the column, flow was directed into a variable wavelength UV detector set at 278 nm. Flow was then directed into the source of an Advion Expression LCMS (Advion Inc., Ithaca, NY, USA) and electrospray ionization (ESI) mass spectrometry was performed in negative ionization mode using selected ion monitoring with a scan time of 50 msec for each of eight polyphenol masses of interest. Capillary temperature and voltages were 300 C and 100 V, respectively. ESI source voltage and gas temperature were 2.6 kV and 240 C respectively. Desolvation gas flow was 240 L/h. Liquid chromatography (LC) and CMS instrumentation and data acquisition were controlled by Advion Mass Express software. Identities of polyphenols in bean samples were confirmed by comparison of *m*/*z* and LC retention times with authentic standards. No “internal” standards were used. External calibration standards were used for all polyphenolic compounds known to affect Fe bioavailability. 

### 2.6. In Vitro Digestion

The in vitro digestion protocol was conducted as per an established in vitro digestion model [12]. Briefly, exactly 1 g of each sample was used for each sample digestion. To initiate the gastric phase of digestion, 10 mL of fresh saline solution (0.9% sodium chloride) was added to each sample and mixed. The pH was then adjusted to 2.0 with 1.0 mol/L HCl, and 0.5 mL of the pepsin solution (containing 1 g pepsin per 50 mL; certified > 250 U per mg protein; Sigma #P7000) was added to each mixture. The mixtures were under gastric digestion for 1 h at 37 °C on a rocking platform (model RP-50, Laboratory Instrument, Rockville, MD, USA) located in an incubator. After 1 h of gastric digestion, the pH of the sample mixture was raised to 5.5–6.0 with 1.0 mol/L of NaHCO_3_ solution. 2.5 mL of the pancreatin–bile extract solution was added to each mixture. The pancreatin–bile extract solution contained 0.35 g pancreatin (Sigma #P1750) and 2.1 g bile extract (Sigma #B8631) in a total volume of 245 mL. The pH of the mixture was then adjusted to approximately 7.0, and the final volume of each mixture was adjusted to 15.0 mL by weight using a salt solution of 140 mmol/L of NaCl and 5.0 mmol/L of KCl at pH 6.7. At this point, the mixture was referred to as a “digest”. The samples were then incubated for an additional two hours at 37 °C, at which point the digests were centrifuged, and supernatants and pellet fractions collected and transferred to tubes for analysis. Three independent replications of the in vitro digestion procedure were carried out for all of the food samples. For some samples, as noted in the specific results section, Fe bioavailability was assessed in both the presence and absence of ascorbic acid (AA). The AA was added to the digests at the start of the gastric digestion phase at a concentration of 10 µmol/L. This treatment has been shown to expose some additional differences between samples and thus provides further information on the matrix of the digest.

### 2.7. Statistical Analysis

Data were analyzed using the software package GraphPad Prism (GraphPad Software, San Diego, CA, USA). Data were analyzed using analysis of variance incorporating normalization of variance, if needed, and Tukey’s post test to determine significant differences (*p* < 0.05) between groups. Unless noted otherwise values are expressed as mean ± standard error of the mean (SEM); *n* = 3 independent replications.

## 3. Results

### Compositional Analyses of Beans, Potato and Rice Samples

Iron and phytic acid levels for the ground bean, potato and rice samples used in experiments one and two are supplied in Table 1. It is important to note that measurement of the Fe content of the bean samples can vary substantially depending on the sub-sampling of the overall harvest. For example, as shown in Table 2, from the same harvests of these lines we measured the Fe level in a separate sub-sample to be 59.9 µg/g in the normal bean and 96.9 µg/g in the high Fe variety. A previously published animal trial using the same harvest of these two lines yielded values of 58 and 106 µg/g for the normal and high Fe varieties, respectively. From experience, we have found that such variation in Fe content is quite common among sub-samples of bean harvests and is simply due to variance in the individual bean Fe content. Thorough mixing of the harvest does not negate this variance; however, the grinding of the sample for analysis makes the sample homogenous for Fe content, with variance of less than 5% between replicates of the ground sample. The amount used for the sub-sampling of the bean harvests of the bean samples should therefore be substantial whenever practical to do so. Based on our observations, we recommend a sub-sample of 200–300 g from thoroughly mixed larger batches, such as 5–10 kg.

Iron analysis of cotyledon, seed coat and embryo fractions demonstrated that the increase in Fe content was consistent across all of the major fractions.

Polyphenolic compounds that are known to influence Fe uptake or that contrasted significantly between the normal and high Fe lines are summarized in Table 3. It is important to note that although other molecular weights were evident in the analyses, only compounds that could be identified are included. Based on previous studies, kaempferol and glycosides thereof are possible Fe uptake promoters, as are catechin and 3,4 di-hydroxybenzoic acid. Inhibitors of Fe uptake are quercetin and procyanidin B [12]. 

The results in Table 4 represent a summary of Fe bioavailability, Fe content, and Al and Ti levels, which are indicators of soil contamination. The green vegetables, cabbage and carrots, tomato sauce, and sombe (a dish with cassava leaves) all appear to have some level of soil contamination. All of these menu items were samples received directly from the cafeteria meal plan of the parallel human study.

The results of Experiment 2 show two significant effects. First, the addition of rice to beans lowers the Fe bioavailability, eliminating the increase in Fe uptake from the high Fe bean. The addition of potato increases the overall Fe uptake from the meal. In both combinations, the bean is the major source of Fe; however, the potato does contribute more Fe to the food matrix relative to rice. For each condition, and it is important to note that the amount of rice or potato is the same as that published in a human study where these lines of beans were evaluated. The second effect shown in Experiment 2 is that the reduction of phytate results in a decrease in Fe uptake. This occurs for both the normal and high Fe beans when evaluated alone or in combination with potato.

**Figure 1:**
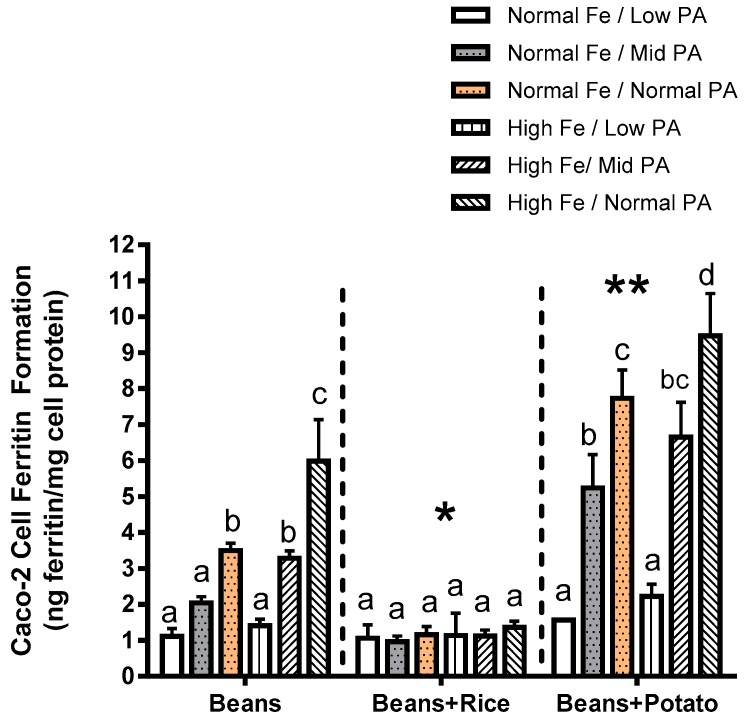
Experiment 2. Iron uptake as measured by Caco-2 cell ferritin formation from beans pre-treated with phytase. Values represent Fe uptake from normal or high Fe beans with normal phytate (PA; 100%) levels, or PA levels reduced to low (34%) or mid (68%) of the normal PA content. Bar values within food combination groups with no letters in common are significantly different (*p* < 0.05). A single asterisk indicates significant inhibitory effect (*p* < 0.05) of the addition of rice, whereas the double asterisk indicates significant promotional effect of the addition of potato.

**Figure 2:**
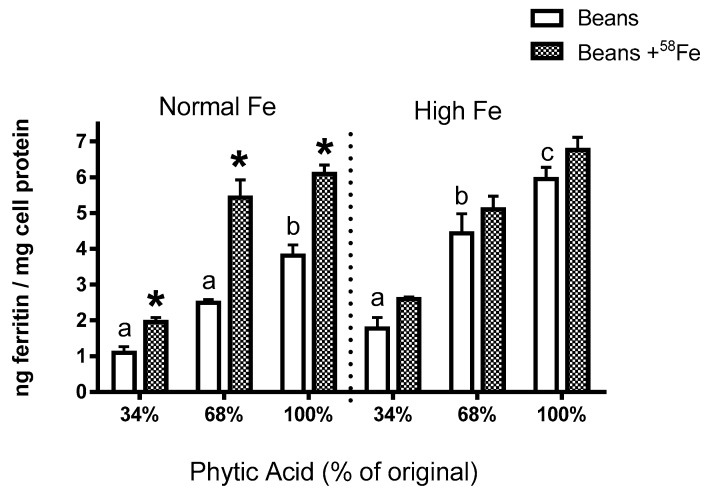
Experiment 3. Caco-2 cell Fe uptake as measured by ferritin formation from normal and high Fe beans that were extrinsically labelled with ^58^Fe as per a published human study using the same harvest of these bean varieties [9]. Values are mean ± SEM, *n* = 3. Bars with no letters in common indicate significant difference (*p* < 0.05). Asterisks indicate significantly more ferritin formation relative to samples without the ^58^Fe label.

**Figure 3:**
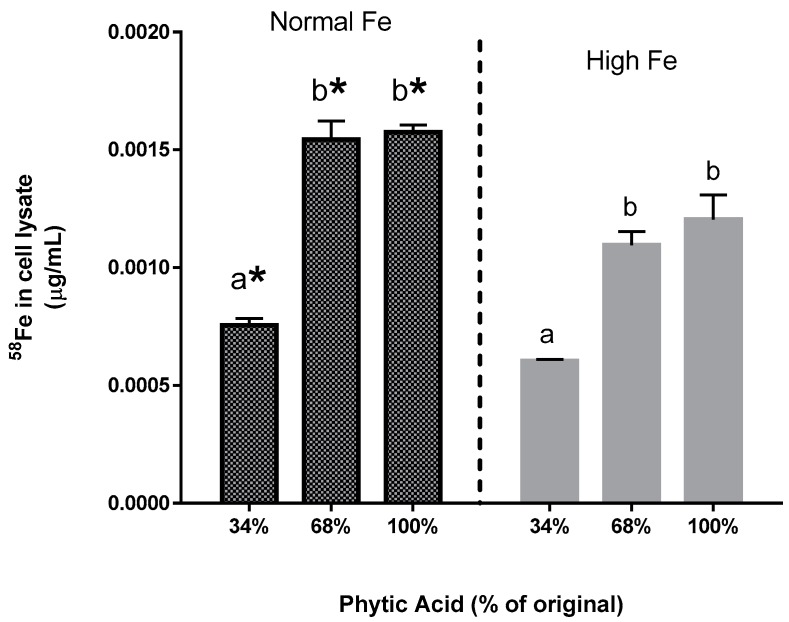
Experiment 4. Caco-2 cell ^58^Fe content following exposure to in vitro digests of normal and high Fe beans extrinsically labelled with ^58^Fe. Values are mean ± SEM, *n* = 3. Bars with no letters in common indicate significant difference (*p* < 0.05). Asterisks indicate significantly more ^58^Fe (*p* < 0.05) relative to the same phytate content in the high Fe beans.

The results in Experiments 3 and 4 are from the same series of experiments. In these studies, efforts were made to replicate the conditions of a human absorption study where the normal and high Fe beans were treated with phytase, and the labelled extrinsically with ^58^Fe [9]. Similar to the results shown in Figure 1, the high Fe bean resulted in significantly more ferritin formation (Fe uptake) than the normal bean, regardless of the phytate content. The addition of the ^58^Fe resulted in significantly higher ferritin formation (iron uptake) from the normal beans but not from the high Fe beans (Experiment 3; Figure 2). Direct measurement of ^58^Fe in the Caco-2 cells showed significantly more ^58^Fe uptake from the normal Fe beans vs the high Fe beans (Experiment 4; Figure 3). Decreased phytate content was associated with decreased ^58^Fe uptake, exhibiting similar pattern of response in Fe uptake as measured via Caco-2 cell ferritin formation.

## 4. Discussion

The in vitro methodology for measuring Fe bioavailability utilized in the present study was first published in 1998 [3]. Since then it has been applied to estimate Fe bioavailability from many food products [13], forms of Fe [14], diet plans [15] and staple food crops [16]. It has been shown to be a robust model, capable not only of diverse application, but also able to handle high throughput [4,6]. Over the past few years, it has been shown to predict the overall outcome of two published human efficacy studies of Fe biofortification and the parallel animal studies [7,8,10,17,18]. A recent review article demonstrates that when coupled with a poultry feeding model, it can now be seen as an established and well-validated approach for the development of Fe-biofortified crops and food products [7]. 

The observations of the present study clearly suggest that several factors should be considered when evaluating and advancing Fe-biofortified beans for either human study or release to farmers. First, it is important to consider how other foods commonly consumed with beans can affect the Fe bioavailability. For example, Experiment 2 indicates that the consumption of beans with rice negates the nutritional benefits of the higher Fe content, whereas the consumption of beans with potato appears to enhance the Fe uptake from the meal. Consumption of beans with rice or potato has been documented as common throughout countries such as Rwanda and other African nations. These combinations were used as test meals in human absorption studies of these same lines of beans, yet it appears that potential differences in these meal combinations were not considered by the investigators [9].

In the above-mentioned human studies, ^58^Fe was added extrinsically to the meal, at levels equalling 11.9% of the total Fe for the normal Fe beans, and at 7.6% of the total Fe for the high Fe variety [9]. Also, in this human study the investigators modified the levels of phytic acid in the bean samples by pre-treating the beans with phytase, claiming to achieve >95% reduction of phytate content. The present study mimicked these treatments using the exact same lines of beans, and used what should be a very effective phytase treatment, but only achieved a maximum decrease in phytate content of 66%. We also explored additional conditions such as increased enzyme concentration and duration of exposure to phytase, but no additional decrease in phytate was observed. Thus, we do not know how the other group was able to achieve almost complete removal of the phytate. This would be of concern as more prolonged treatment could also be more likely to alter other factors in the bean samples, such as polyphenolic profiles that would confound the results relative to the native phytate and polyphenol profiles of these lines.

The present study yields some key observations on Fe bioavailability with the reduction of phytate content. First, the decrease in phytic acid resulted in a surprising decrease in Fe uptake by the Caco-2 cells (Experiments 2–4), an observation which is in contrast to the claim from the human study that dephytinization increased Fe uptake. One possible explanation for the in vitro decrease in Fe uptake could be that the decrease in phytate facilitated greater opportunity for the seed coat polyphenols to complex the Fe and inhibit Fe bioavailability. However, with the addition of ^58^Fe to the bean samples, there is an interesting observation that could explain the apparent difference between the human study results and this in vitro study when phytate content is decreased. Consider the following: the addition of the extrinsic ^58^Fe was clearly associated with an increased ferritin formation effect (i.e., Fe uptake) in the normal beans but not in the high Fe beans (Experiment 3). Indeed, for the normal bean samples, where phytate values were reduced to 34% and 68% of the natural content, the addition of the ^58^Fe approximately doubled the Caco-2 cell Fe uptake. A similar trend was evident in the high Fe beans although the difference was not statistically significant. The lesser effect in the high Fe bean samples is probably due to the increased intrinsic Fe content or possibly to other factors such as the differences in polyphenol content (Table 3). Direct measurement of the ^58^Fe in the Caco-2 cells also indicates that the increased ferritin formation is due to the extrinsic ^58^Fe (Experiment 4). Indeed, the addition of an extrinsic Fe source at an amount equal to 11.9% of the total Fe approximately doubles the Caco-2 cell ferritin formation. Taken together, these observations indicate that the uptake of the extrinsic Fe was much greater than that of the intrinsic Fe of the bean. In other words, the extrinsic ^58^Fe has a different bioavailability relative to the intrinsic Fe of the bean and is clear evidence of incomplete isotopic exchange and equilibration of the extrinsic label. It should be noted that previous studies have shown that the cotyledon cell walls of beans are a potential barrier to Fe uptake from a bean meal, and could also be a barrier that prevents equilibration of the extrinsic Fe with the intrinsic Fe [19]. 

As noted in studies decades ago, extrinsic labelling of food Fe is dependent upon the assumption of complete isotopic exchange and equilibration [20]. Hence, the above observation indicates that extrinsic labelling of the bean samples as conducted in the human study would yield inaccurate measurement of Fe bioavailability from the beans under these feeding conditions. It is therefore entirely possible that in the human study, the decrease in phytate did indeed result in less overall Fe uptake, an effect that would have been masked due a flawed assumption of complete extrinsic label equilibration. The human study depends entirely upon this assumption, whereas the in vitro model does not.

In consideration of the above, it is also important to note that the human study [9] did not separate out the effects of rice versus potatoes on Fe uptake from the meals; thus, the Fe uptake in the human study represents the combined effects of rice and potato on isotopic Fe uptake. This combined food effect, plus the fact that the human study did not simply measure Fe uptake from just the beans, limits further useful comparison of the in vitro and human results. Certainly, future studies should follow up on the individual effects of rice and potatoes on bean Fe bioavailability as it negates the benefit of a crop in a given food system. In regards to comparing the human absorption study with the in vitro model, the question that remains is which approach is more accurate and effective at evaluating the Fe bioavailability of beans? The human model is based on an assumption that has seen a fair share of criticism and doubt by other scientists [11,20,21]; whereas, the in vitro approach bears the caveat of simply being an in vitro model, albeit one that is now validated by direct comparison to human efficacy and animal feeding trials [7]. 

The present study also demonstrates that the high Fe beans also contained higher levels of uptake-inhibiting polyphenols in the seed coats (Table 2). For these bean varieties, the inhibitory compounds of most significance are quercetin and quercetin-3-glucoside. These compounds have been shown to be inhibitory of Fe uptake, even when potential promoting polyphenols, such as kaempferol (and glycosides thereof) are present in greater amounts [12]. Increased inhibitory polyphenols were also demonstrated in high Fe black beans and high Fe pearl millet [22,23]. This consistent association of enhanced Fe content with greater polyphenol levels reinforces the need to perform bioavailability assessments of lines targeted for biofortification. Moreover, the finding of increased inhibitory polyphenols with increased Fe content is an example of how breeding solely for enhanced Fe content could result in a misdirection of breeding. Such a misdirection could result in an end product with no nutritional benefit, despite enhanced content, or one that achieves a less than maximal benefit. It is important to note that the compounds listed in the present study represent the ones that are currently known to be among the list of polyphenols found in bean seed coats and are known to influence Fe bioavailability. There may be others, but this list is simply based upon what has been identified and characterized from previous work that utilized the Caco-2 cell bioassay and LC-MS technology [12].

Evaluation of the results presented on the menu items of the Rwandan human trial (Experiment 1) must consider that these results are based on the dry weight of the menu items. We do not have data for the water content of these menu items. In general, it could be expected that for items such as tomato sauce the water content could be 90–95%, perhaps slightly less for items such as green vegetables, cabbage, carrots, and banana. The potato items could be of key nutritional interest, depending on their water content, as the Caco-2 cell ferritin formation and Fe content values were relatively high. The green vegetables appear to be contaminated with soil Fe as Fe, Al and Ti values were very high and ferritin values were low, measurements that are clear indicators of the presence of soil Fe. The occurrence of the items on the menu are also potentially important; and although it was beyond the scope of the present study, it should be noted that the experimental approach outlined in this manuscript could certainly be applied to evaluate the meal plan in greater detail, provided that information such as water content and consumption rate are available.

## 5. Conclusions

The present study clearly shows that the high Fe bean variety delivers more absorbable Fe relative to the normal bean variety. Such observations agree with previous in vitro, animal and human studies of these particular varieties and harvests [8,10]. Indeed, the level of validation of this in vitro approach is now quite extensive; hence, observations generated from its proper application should be confidently applied to direct nutritional studies and bean breeding. In addition, the results from the present study provide more evidence that the assessment of the Fe bioavailability of beans via extrinsic labelling yields potentially inaccurate information.

## Figures and Tables

**Table 1:** Iron (Fe) and phytic acid (PA) content of beans for Experiments 1 and 2. The rice and potato samples were used in Experiments 3 and 4 ^1^.

Food Sample	Fe (µg/g)	Phytic Acid g/100 g	Molar Ratio PA:Fe
Normal Fe bean	47.5	1.21	22:1
High Fe bean	82.5	1.52	16:1
Potato	19.1	0.33	14:1
Rice	2.2	0.14	53:1

^1^ Values represent the average of three replicate measurements. Range of variance in measurement was <5% for all samples.

**Table 2:** Iron content of cotyledon, embryo and seed coat of the normal and high Fe beans ^1^.

Bean Variety	Cotyledon Fe (µg/g)	Embryo Fe (µg/g)	Seed Coat Fe (µg/g)	Total Fe (µg/g)
Normal	51.5 (78.3%)	67.1 (1.2%)	156.4 (20.6%)	59.9
High Fe	81.6 (75.7%)	103.3 (1.5%)	255.9 (22.8%)	96.9

^1^ Values in parentheses represent the percent of the total Fe present in this fraction of the whole bean. Values represent the average of three replicate measurements. Range of variance in measurement was <5% in all samples.

**Table 3:** Polyphenolic profile of the seed coat of the normal and high Fe beans ^1^.

Compound	Molecular Weight (*m*/*z*)	Normal Bean	High Fe Bean
Kaempferol-3-glucoside	447.0926	281	475
Kaempferol derivative	489.1030	108	251
Catechin	289.0710	237	182
AzIV	1083.5415	135	32
Kaempferol	285.0393	50	101
Procyanidin B	577.1348	106	84
Querctin-3-glucoside	463.0874	ND	81
Kaempferol-3-sambubioside	579.1354	ND	25
3,4-dihydroxybenzoic acid	153.0180	19	24
Quercetin	301.0347	ND	19

^1^ Values for compound levels represent ion intensity, and therefore represent only relative differences in concentration. “ND” = non-detected.

**Table 4:** Experiment 1. In vitro Fe bioavailability, and mineral (Fe, Al, Ti) content of menu items from human efficacy trial of normal and high Fe beans [8] ^1^.

Menu Item	Occurrence on Menu (%)	Fe Bioavailability (ng Ferritin/mg Cell Protein)	Fe (µg/g)	Al (µg/g)	Ti (µg/g)
Normal bean	100	5.01 ± 0.2	47.5	5.2	0.2
High Fe bean	100	5.9 ± 0.2	82.5	5.9	0.4
Tomato sauce	100	24.2 ± 1.1	120.8	76.8	0.5
Rice	85	0.9 ± 0.1	4.7	1.7	0.2
Cooked rice with curry	unknown	1.7 ± 0.3	6.5	14.5	0.2
Cabbage + carrots	43	9.5 ± 0.3	80.0	80.2	1.8
Green vegetables	43	1.5 ± 0.3	312.7	448.1	14.4
Potato chips	28	20.9 ± 1.1	25.4	8.0	1.2
Banana chips	unknown	1.8 ± 0.2	10.5	2.5	0.4
Fried sweet potatoes	14	7.3 ± 1.1	22.1	10.0	0.6
Cassava bread	14	5.69 ± 0.55	22.2	22.8	1.3
Macaroni	14	2.04 ± 0.26	34.3	5.8	0.3
Sombe	14	1.40 ± 0.21	81.0	168.1	0.8
Fried Irish potatoes	7	25.51 ± 1.33	30.4	8.6	1.3
Boiled Irish potatoes	7	41.77 ± 2.82	37.7	3.5	0.6
Banana with tomato sauce	14	1.91 ± 0.04	20.0	11.7	0.7

^1^ Fe bioavailability values are mean ± standard error, *n* = 3. Mineral content values are the mean of three replicates.
